# Foundations for the Successful Establishment of a Multidisciplinary Orthoplastic Surgery Team

**DOI:** 10.1055/a-2867-5035

**Published:** 2026-07-02

**Authors:** Elliot L.H. Le, Evan Haas, Marlie H. Fisher, Jerry H. Yang, David W. Mathes, Matthew L. Iorio, Mark A. Greyson

**Affiliations:** 1 Plastic and Reconstructive SurgeryUniversity of Colorado Anschutz Medical Campus School of MedicineAurora, ColoradoUnited States

**Keywords:** lower extremity reconstruction, microsurgery, orthopedic trauma, lower, extremity trauma, orthoplastische Chirurgie, Rekonstruktion an der unteren Extremität, Mikrochirurgie, Trauma der unteren Extremität, Orthopädische Traumatologie

## Abstract

Orthoplastic surgery, a multidisciplinary approach to addressing complex, traumatic reconstruction, has grown significantly since its emergence over three decades ago. Its success in improving both functional and reconstructive outcomes has led to an increased demand for orthoplastic surgeons and the development of dedicated limb salvage programs at institutions around the world. This review outlines the development of a multidisciplinary orthoplastic surgery program at a Level 1 trauma center in the United States, which serves as a major tertiary referral hub for complex extremity trauma and reconstruction. Central to the success of this practice is collaboration between multiple surgical and medical subspecialists. Coordination of our program is enhanced through center-supported staff such as nurse navigators and practice managers. The goal of this review article is to highlight the foundations that our program is built on and provide a framework for other institutions to emulate when establishing an orthoplastic surgery program.

## Zusammenfassung

Die orthoplastische Chirurgie, ein multidisziplinärer Ansatz zur Behandlung komplexer traumatischer Rekonstruktionen, hat seit ihrem Entstehen vor über drei Jahrzehnten erheblich an Bedeutung gewonnen. Ihr Erfolg bei der Verbesserung sowohl funktioneller als auch rekonstruktiver Ergebnisse hat zu einer steigenden Nachfrage nach orthoplastischen Chirurginnen und Chirurgen sowie zur Entwicklung spezialisierter Extremitäten-Erhaltungsprogramme an Institutionen weltweit geführt.

Diese Übersichtsarbeit beschreibt den Aufbau eines multidisziplinären orthoplastischen Chirurgieprogramms an einem Level-1-Traumazentrum in den Vereinigten Staaten, das als bedeutendes tertiäres Überweisungszentrum für komplexe Extremitätentraumen und Rekonstruktionen dient. Zentral für den Erfolg dieses Programms ist die enge Zusammenarbeit zwischen mehreren chirurgischen und medizinischen Subspezialitäten. Die Koordination des Programms wird zudem durch zentrumsunterstütztes Personal wie Pflege-/Fallmanagerinnen und -manager (Nurse Navigators) sowie Praxismanagerinnen und -manager verbessert.

Ziel dieses Übersichtsartikels ist es, die Grundlagen darzustellen, auf denen unser Programm aufbaut, und einen Rahmen zu bieten, an dem sich andere Institutionen beim Aufbau eines orthoplastischen Chirurgieprogramms orientieren können.

## Introduction

The term “orthoplastic surgery” was first widely used by Dr. L Scott Levin in 1993 to describe an approach to extremity reconstruction that incorporated the core principles of both plastic and orthopedic surgeries 1 . Orthoplastic surgery emphasizes the importance of a multidisciplinary surgical approach originating in the care of extremity trauma and has now extended to managing complex oncologic defects, chronic wounds and vasculopathic lower extremity conditions.

Orthoplastic surgery relies on collaboration and coordination across many other disciplines, including vascular surgery, interventional radiology, rehabilitation medicine, oncology, infectious diseases, rehabilitation services, and social work. Specialists must work in concert, as these injuries often require the meticulous management of infection burden, vascular compromise, tissue loss, and extensive postoperative rehabilitation to optimize surgical salvage outcomes.

A pivotal study by Godina et al marked a critical development in the evolution of orthoplastic surgery, demonstrating that early soft tissue coverage, preferably within 72 hours of injury, significantly reduces the likelihood of developing major complications, including flap failure and infection 2 . The advent of negative pressure therapy and later investigations of the timing of soft tissue coverage reinforce Godina’s findings and have further suggested that successful surgical outcomes may still be achieved when tissue coverage is performed beyond the 72-hour window 2 3 4.

In the past 20 years, this discipline has seen rapid growth and global interest. In the United States, the emergence of orthopedic and plastic surgeons specializing in limb salvage has led to a concentration of expertise with expansive regional referral networks. In addition to clinical interest, research has also increased in this space, growing from three “orthoplastic surgery” publications indexed in PubMed in 2010 to 46 articles in 2024. Current research includes multicenter efforts to establish standards for limb salvage centers of excellence, support eventual accreditation, and reaffirm established principles 5.

This article outlines the development of our institution’s orthoplastic surgery program and the establishment of a regional center of excellence for managing complex lower extremity trauma and diseases.

### The reconstructive plastic surgeon

The most important piece to establish in this multidisciplinary team is to find surgeons with expertise and interest in both plastic and orthopedic limb reconstruction. The reconstructive plastic surgeon must possess a broad lower extremity skill set and also be capable of integrating orthopedic priorities, including skeletal stability, alignment, and gait mechanics when planning durable soft tissue coverage that supports musculoskeletal reconstruction.

The goal of this unique service line is to restore the form and function of an open orthopedic injury. It is insufficient to simply cover a wound with pedicled or free tissue without considering the subsequent steps for functional reconstruction. Plastic surgeons are often the most equipped and interested in this subset of reconstruction and typically have formal training in either hand surgery or microsurgery. Uniquely, plastic hand surgeons are trained in plastic and orthopedic surgery concepts in the functional reconstruction of upper extremities and many of those skillsets and surgical interests translate well to the lower extremity – free tissue transfer, pedicled flaps, skeletal stabilization, musculotendinous reconstruction, and peripheral nerve surgery 6.

### Interdisciplinary team

It is critical to assemble a team whose collective expertise covers every aspect of complex extremity reconstruction to grow and maintain a successful limb salvage practice. From this principle, we have been able to build a truly multidisciplinary team capable of complex orthoplastic limb reconstruction. At the core of our program are vascular surgeons who assess and restore vascular flow; orthopedic trauma specialists who stabilize complex fractures, nonunion and malunions; and plastic surgeons with dedicated training in limb reconstruction, microsurgery, and peripheral nerve repair. In line with our center’s Level 1 designation, we have established a dedicated inpatient Lower Extremity Plastic and Reconstructive Surgery service. This team is composed of hospital-supported plastic surgeons on 24/7 call and is supported by orthopedic surgeons to provide “orthoplastic coverage.” Additionally, to support involvement of plastic surgery following these cases, our center also supports an inpatient physician assistant who assists with complex wound care and flap monitoring.

Beyond the orthopedic and plastic surgeons, infectious disease physicians guide a targeted antimicrobial strategy, musculoskeletal radiologists provide the real-time visualization of anatomy to delineate hard and soft tissue pathology, and vascular surgeons help intervene on pathological arterial anatomy to optimize distal blood flow. Postoperatively, physical medicine and rehabilitation physicians provide patients with a pathway for functional recovery. Moreover, social workers coordinate resources for our patients and help address the psychosocial barriers of complex and expensive medical care and access to rehabilitative services.

A devoted practice manager and a nursing specialist or “nurse navigator” are also vital for the success of our limb salvage practice. Our practice manager orchestrates the use of shared operating rooms across multiple teams, many of which require unique instruments, setup, and staff. The nurse navigator serves as the liaison between clinical teams and ancillary services and is largely responsible for minimizing logistical errors, which may delay tissue coverage or prolong ischemia time. Furthermore, and in more recent efforts to improve efficiency, our practice manager has begun scheduling blocked operating room time specifically for free flaps to minimize delays in coverage. This dedicated, blocked operating room time is reserved for flap reconstruction, alternating Mondays and Tuesdays, with plans to expand this service as volume grows.

As our multidisciplinary team grew in both size and expertise, it also allowed us to broaden our surgical indications beyond the repair of traditional trauma. Within these expanded indications were patients with acute (“hot”) and chronic (“cold”) traumatic injuries, complex regional pain syndrome, oncologic defects, and patients with vasculopathic conditions. As our service line grew busier and case complexity grew, seamless communication across the care team became more critical.

### Team communication

Our program is bolstered by multiple levels of robust communication. Each week, our team meets to review complex cases, in the same manner as tumor boards. These meetings include orthopedic trauma, plastic surgery, vascular surgery, infectious diseases, rehabilitation medicine, and radiology, along with social work and psychiatry as needed. Relevant imaging is reviewed as a group and operative plans, postoperative recovery strategies, and anticipated social challenges are discussed. Because there is rarely a stepwise or standard reconstructive algorithm in orthoplastic reconstruction, it is necessary to discuss each case from multiple perspectives to ensure that critical considerations are not missed.

In cases of acute trauma, we complement our weekly formal meetings with real-time communication where the orthopedic team often initiates communication to rapidly involve our plastic surgeons when extremity trauma patients are admitted. This early involvement is crucial, as minimizing “culture to coverage” time, the interval between adequate debridement and definitive reconstruction, is required to increase chances of operative success 2 3 4 . We have developed a system whereby orthopedic surgery will inform the plastic surgery service of the patient in question and coordinate a subsequent operative time where both teams can be available to assess the wound intraoperatively and determine a reconstructive plan 7 . One tactic that has improved our service’s efficiency greatly is the consolidation of operating rooms. Orthopedic and plastics cases are routinely placed in adjacent operating rooms when feasible, and allow for real-time collaboration, which is particularly valuable for plastic surgeons to obtain a “first look” at open fractures.

This unified communication and coordination are paramount as many studies have demonstrated the importance of expeditious coverage of high-grade open injuries 8 9 10 . By communicating early, we can utilize technology such as negative pressure therapy, which has been proven to extend the timeline of reconstruction and even allow orthopedic surgeons to definitively fixate the osseous injury prior to soft tissue coverage 11 12 . With extra time, surgeons from both the orthopedic and plastic surgery teams can coordinate the sequence of procedures required for a “hot” trauma patient including external fixation, internal fixation, and soft tissue coverage.

From the patient’s perspective, our nurse navigators are the central hub of communication and care coordination across our program and with the patients. Their focus is especially important in the follow-up care after initial reconstruction and hospitalization. In addition to their clinical work, they coordinate clinic visits, imaging, and perioperative evaluations across multiple specialties. They are the primary contact for patients and their families, updating them on scheduling, aftercare, and relay patient concerns back to the rest of the care team. By being a point of contact for patients throughout their entire reconstructive journey, they transform a seemingly complex surgical program into a personalized and patient-centered experience.

### Our center and referral network

**Fig. 1:**
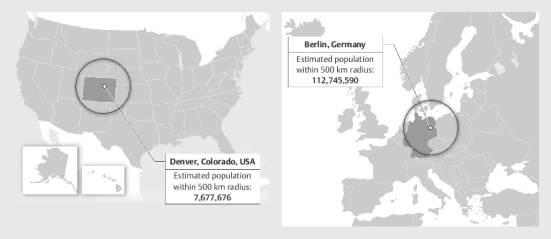
Geographic comparison of referral catchment areas (map courtesy of Vemaps). A 500-mile radius around our center in Colorado (left) is compared with a referral network catchment area in Germany, centered on Berlin (right). This illustrates differences in geographic scale and patient referral distribution between the U.S. and European systems.

Our health system is a large tertiary referral center and is designated as a "Level 1 Trauma Center" – the highest designation recognized by the American College of Surgeons – in the eastern Colorado metropolitan region. A Level 1 center acts as a regional resource that provides all aspects of care for all types of injuries from initial resuscitation to rehabilitation, provides ongoing education on trauma management, and maintains 24/7 availability of in-house surgical specialists. Unlike Level 2 and 3 trauma centers, which generally stabilize patients with varying degrees of trauma, Level 1 centers serve as the final resource for complex reconstruction and rehabilitation. Due to this designation, our orthopedic and plastic surgeons receive referrals daily for all types of injuries and reconstructive problems from other hospitals. Our center is one of a few Level 1 health systems within a 500-km radius of Denver, Colorado which is equal to 7.7 million people. For comparison, as shown in Fig. 1, a similar geographic area juxtaposed over Berlin, Germany, encompasses 112 million people, including non-German citizens 13 . Our region sees a unique range of traumatic injuries ranging from high energy motor vehicle collisions and gunshot wounds to low energy etiologies such as machinery and agricultural injuries 14 15 16 . Thus, we frequently will see severe limb injuries requiring orthoplastic reconstruction.

### Surgical model

While our institution draws on multiple relevant practice guidelines, our practice primarily relies on guidelines from the British Orthopaedic Association and the British Association of Plastic, Reconstructive and Aesthetic Surgeons (BAPRAS) and British Orthopaedic Association (BOA), which have become the widely adopted standards for the management of open lower extremity fractures 17 . These standards emphasize formal orthoplastic collaboration, operative planning, and timely definitive fixation and soft tissue coverage. We use this framework as a practical model when designing and refining the operational structure of our inpatient pathway.

To begin the cascade of orthoplastic reconstruction at our institution, the orthopedic surgery team will consult our two senior hand surgeons and director of orthoplastic surgery in nearly all cases of open lower extremity fractures at the time of injury or during the initial investigative surgery. The first operation is an opportunity for both teams to complete a full intake of the extent and severity of the injury, account for critical and expendable structures, and determine the best course of action for each structure – skeletal, musculotendinous and soft tissues. Tissue is debrided to viable bleeding edges careful to not excise critical structures unless necessary, and tissue and bone cultures are sent in the setting of open and contaminated injuries. The wound is temporarily closed with negative pressure dressings and the orthopedic surgery team will plan the next surgery for 24 to 72 hours.

Subsequent surgeries will be planned with the entire multidisciplinary team. The orthopedic surgery team will confirm that the osseous and soft tissue structures are free of bacterial contamination, schedule repeat debridement as necessary, and then plan fixation whether it is definitive internal fixation or external fixation. The plastic surgeon will have the foresight to envision the final reconstruction, where to obtain missing components, and how best to time each step of the reconstruction. Depending on the defect characteristics, we prioritize local options such as pedicled or propellor flaps when they are sure to provide adequate coverage 18 19 . However, coverage is dictated by the wound’s requirements and should follow the reconstructive ladder, which allows our teams to balance both the durability and the aesthetic outcome, while also accounting for the possibility of future orthopedic procedures.

**Fig. 2:**
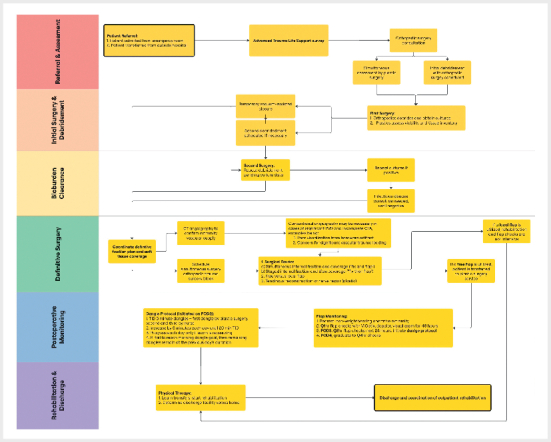
A flowchart of the inpatient orthopedic trauma management on the orthoplastic surgery service. The pathway begins with patient referral and initial evaluation, followed by staged debridement, culture-directed infectious disease management, and vascular imaging as indicated. Definitive surgery is coordinated jointly by orthopedic and plastic surgery teams, with simultaneous or staged fixation and soft tissue coverage. Postoperative care includes flap monitoring, rehabilitation, and structured discharge planning, tailored to functional and recovery needs.

Definitive reconstruction may follow simultaneous or staged reconstruction. In simultaneous reconstruction, sometimes referred to as a “fix and flap” model, definitive internal fixation and flap coverage occur during the same operation after adequate debridement, with shared efforts from orthopedic trauma and plastic surgery 20 . In staged reconstruction, referred to as “fix then flap” reconstruction, definitive fixation is followed by delayed flap coverage in a separate operation. Staged reconstruction is typically performed at our institution to accommodate for repeat debridement, bioburden clearance, and operating room coordination. Although delayed definitive coverage has been associated with higher rates of infection, the complexity and significant contamination of the injuries we treat, along with logistical constrains often necessitate a staged approach 21 . Our inpatient pathway, as illustrated in Fig. 2 , is designed to support either sequence, with an emphasis on minimizing delays while being conscious of the wound’s reconstructive requirements.

In the event of distal or large wounds, our team has expertise with superthin and suprafascial perforator flaps, and these are typically our reconstruction method of choice 22 23 24 . Other aspects of lower extremity reconstruction that are underappreciated include restoration of gliding surfaces that can withstand shear forces of a weight bearing and moving extremity as well as the durability of the reconstructed limb. In addition to durability, we will consider the simultaneous reconstruction of tendinous or osseous structures with chimeric tendons and soft tissue flaps 25 26 27 . In preparation for soft tissue reconstruction, we will obtain arteriography of the extremity to examine the vascular supply. A CT arteriogram with MIPS sequences is typically sufficient; however, in the setting of arterial injury with concomitant peripheral arterial disease, or extensive artifact from hardware, a conventional arteriogram is sometimes indicated. The findings from this study are critical for preoperative planning 28.

As stated previously, our cooperation goes beyond communication as the orthopedic and plastic surgery teams’ schedulers will consolidate operating room schedules. We often will schedule patients’ final orthopedic surgery and soft tissue reconstruction under the same operation in the orthopedic surgery team’s operating room and block time as the first case of the day to allow both teams ample time and space to complete the patient’s reconstruction. We will also coordinate with our anesthesiology colleagues for regional continuous blocks to address postoperative pain and help decrease the sympathetic response in the extremity in anticipation of free flap reconstruction 29 .

Prior to free tissue transfer reconstruction of the patient’s extremity, all their care will be directed by the orthopedic surgeon and their residents. After the free flap operation, patients will be transferred to the plastic surgery service in the intensive care unit for flap monitoring and progression through a standardized early dangle protocol. The purpose of this is to condition the flap for the increased venous congestion it will experience on a gravity-dependent limb 30 . Flap monitoring includes continuous tissue oximetry monitoring of the flap with ViOptix (Newark, CA), pencil Doppler checks and visual exams by nursing every hour for 48 hours. After 48 hours, nurse flap checks are spaced to every 2 hours for 1 day and every 4 hours afterwards. The dangle protocol starts on postoperative day 2 if there are no issues. The patient will transfer to the side of the bed and dangle their extremity three times/d for 5 minutes each time. The first dangle each day will be done with the plastic surgery team observing the flap’s color, Doppler examination and ViOptix reading. Subsequent dangles will be performed with the patient’s nurse. The dangle duration will increase by 5 minutes each day if the exam is reassuring until the flap can tolerate 20 minutes of dangles three times/d. Reasons for terminating a dangle early include the tissue oximetry level decreasing more than 20% points, absence of arterial and venous signals upon Doppler examination, and visible venous congestion that is moderate to severe. This process is subjective and requires the team and nurses to be experienced in flap checks and exams. If a dangle is ended prematurely, remaining dangles for the day will continue at the previous day’s prescribed length. After a patient reaches the end of the dangle protocol, the patient will then begin working with our physical therapy team to practice transferring, determine the appropriate facility setting for discharge, and start the rehabilitative process. Patients are advised preoperatively that their recovery will require hospital admission for 7–14 days after free flap reconstruction. A flow chart representation of our inpatient workflow is highlighted in Fig. 2 .

### Reframing amputation as “limb-restoring”

In cases where injuries are too severe for limb salvage or limb salvage fails, amputation is considered. If amputation is considered and there is no concern for acute necrotizing soft tissue infection, this decision is preceded by an extensive discussion with the patient and the surgical teams. Our orthopedic trauma surgeons also uniquely specialize in amputee care, including osseointegration, which allows for the direct attachment of a prosthetic attachment to a patient’s skeleton 31 . Recent data from our orthopedic department demonstrate that osseointegration significantly improves the patients’ reported pain and quality of life outcomes in lower extremity amputees 32 . This added specialization has expanded our plastic surgery team’s involvement in limb care as soft tissue reconstruction in patients with osseointegration is different from routine amputation stump closure. In both primary amputations and patients pursuing osseointegration, the plastic surgery team is involved in the closure of the residual limb and attentive management of the soft tissue 33 . In addition to standard postoperative pain regimens, we also offer nerve directed strategies to reduce neuroma formation and phantom limb pain, including targeted muscle reinnervation (TMR) and regenerative peripheral nerve interfaces 34 35 36 37 . For TMR, we use established anatomic nerve distributions and intraoperative exploration to identify the transected major lower extremity nerves and select appropriate recipient motor branches 38 . The extensive research by our team on the benefits of TMR on surgical outcomes and neuroma treatment has guided its use in our practice 36 37 39.

### Expanding scope of practice

Our multidisciplinary limb salvage program started in 2017, and prior to 2021, we had only one extremity plastic surgeon. This surgeon focused most of their care in acute trauma patients as described above. In 3.5 years, our program performed 45 free flaps – 33% ( *n* =15) were acute traumas that were transferred to the orthopedic surgery service for immediate care of open orthopedic injuries. Another 40% ( *n* =18) were patients referred to our center for complex revisions of complications following orthopedic trauma care including nonunion, malunion, chronic osteomyelitis, and unstable soft tissue envelopes. We label this population as delayed trauma patients. These patients are often referred to our center as a revision case with their index reconstruction performed elsewhere. The chronicity of their complication has led to poor soft tissue quality (i.e., unstable scar) that will not withstand the “trauma” of multiple reoperations to revise the underlying skeletal structure. This is especially evident in cases of bony reconstruction such as Masquelet techniques and Ilizarov distraction osteogenesis 40 41 42 43 . Consequently, our service is routinely consulted for soft tissue coverage options in these settings. Flap design and plane of elevation are selected based on the needs of the recipient wound bed and the thickness of the donor site’s subcutaneous tissue. When appropriate, we often utilize fasciocutaneous flaps as we are able to elevate the flap at the desired thickness applicable to the needs of the defect, and once transferred, it can provide a discernable layer that can be re-elevated during subsequent revision surgeries. All 87 free flaps we have performed between 2017 and 2024 are fasciocutaneous flaps with the anterolateral thigh flap being our preferred option (92%, *n* =80).

The natural evolution of our program in addressing more complex reconstructions and consultations is evident in our patient mix following 2021. In 2021, we were able to recruit an additional extremity plastic surgeon and between 2021 and 2024 and were able to complete 42 free flaps – 57% of which were delayed trauma patients. Our program grew more efficient as well, with median length of stay decreasing from 26 to 24 days in acute trauma patients and decreasing from 31 to 20.5 days in delayed trauma patients. Median days from free flap completion to discharge, although grossly unchanged, reflects the careful considerations we make in the postoperative period coordinating rehabilitation needs, home health equipment, and social resources to provide a smooth recovery. In the 7 years, we have lost nine free flaps (10.3%) resulting in four lower extremity amputations (4.6%). Among these nine initial failures, one patient chose to proceed with amputation, and the remaining eight received a second free flap. Two of these patients failed their second flap and underwent amputation while the fourth patient developed chronic osteomyelitis despite a successful soft tissue reconstruction resulting in an amputation.

The next goal of our program is to incorporate the orthoplastic approach in other complex pathologies including orthopedic oncology and vascular surgery and to address chronic wounds. These cases reflect a natural extension of our operative and perioperative capabilities and are now an integral part of our multidisciplinary workflow. This expansion is not without important considerations and a thoughtful rollout. For example, expansion into orthopedic oncology patients will likely involve our extremity plastic surgeons participating in existing oncology multidisciplinary conferences rather than corollary. These patients have complex care needs including radiation oncology consultations and the participation of other surgical services such as vascular surgery, colorectal surgery, spine surgery. We have already begun this process and have appointed one of our extremity surgeons as the liaison between our service lines.

From a research perspective, we look forward to finalizing existing database collection on free flap reconstruction while also expanding our focus beyond this. Free bone flaps, pedicled flaps, peripheral nerve reconstruction, and amputation care (e.g., Osseointegration) are areas that our extremity surgeons have experience in but are not heavily considered by our research teams yet. Finally, quality and process improvements are key to the evolution and success of any team. We have much room for improvement when it comes to postoperative coordination of care as the length of stay after reconstructive surgery should not exceed many more days beyond the completion of a dangle protocol. The American health care system can be a complex system to navigate, but our goal is to find ways to be more proactive in the coordination of patient resources and rehabilitation services after surgery.

In summary, the development of an inpatient multidisciplinary orthoplastic service line for extremity trauma reconstruction can yield a multitude of surgical opportunities. These opportunities then lead to a busy practice for both specialty teams. This niche service line also is integral for any health system that aspires to provide high level tertiary care for a large catchment area. Finally, the development of a streamlined process for acute reconstructions can also be translated into orthoplastic surgery for amputee care, extremity oncologic pathologies, vasculopathic pathologies and chronic extremity wounds.

## Authors

Dr. Elliot Long Hoang Le is a chief resident in the Integrated Plastic and Reconstructive Surgery Program at the University of Colorado School of Medicine, under the guidance of his residency director and senior author, Dr. Mark Greyson. He has authored over 30 peer-reviewed publications examining outcomes in microsurgical limb salvage, flap timing, and peripheral nerve reconstruction and has presented his work at national and international meetings. Following residency, he will pursue a fellowship in reconstructive microsurgery at the Buncke Clinic in San Francisco.
